# ﻿The family Polycentropodidae (Insecta, Trichoptera) in mid-Cretaceous Burmese Amber

**DOI:** 10.3897/zookeys.1134.93999

**Published:** 2022-12-09

**Authors:** Wilfried Wichard, Chunpeng Xu

**Affiliations:** 1 Universität zu Köln, Institute of Biology and its Didactics, Herbert Lewinstraße 2, 50931 Cologne, Germany Universität zu Köln Cologne Germany; 2 State Key Laboratory of Palaeobiology and Stratigraphy, Nanjing Institute of Geology and Palaeontology and Center for Excellence in Life and Paleoenvironment, Chinese Academy of Sciences, Nanjing 210008, China Nanjing Institute of Geology and Palaeontology and Center for Excellence in Life and Paleoenvironment, Chinese Academy of Sciences Nanjing China; 3 University of Chinese Academy of Sciences, Beijing 100049, China University of Chinese Academy of Sciences Beijing China

**Keywords:** Hukawng valley, Kachin amber, *Neureclipsis* cluster, *Polycentropus* cluster

## Abstract

Three described species, *Neureclipsistriangula***sp. nov.**, *Neureclipsisacuta***sp. nov.**, and *Neureclipsisobtusa***sp. nov.**, expand the *Neureclipsis* cluster to six species dominating the Polycentropodidae in Burmese amber. The new species *Plectrocnemiaohlhoffi***sp. nov.** and *Plectrocnemiabowangi***sp. nov.** of the *Polycentropus* cluster add to the comparatively low occurrence of Polycentropodidae in Burmese mid-Cretaceous amber.

## ﻿Introduction

The caddisfly family Polycentropodidae is one of the most diverse in the trichopteran suborder Annulipalpia and is distributed worldwide, today with about 891 extant species (Chamorro and Holzenthal 2011; Morse 2022). The adults can be distinguished from species of all other families by the following combination of characters: ocelli absent in adult; antennae never longer than forewings; maxillary palpi each five-segmented, first two segments short, each shorter than the third or fourth, the fifth longest and annulated; mesoscutum with a pair of rounded setal warts; mesoscutellum with a rounded setal mesal wart; tibial spurs 2–3/4/4; in male genitalia inferior appendages each one-segmented.

According to Oláh and Johanson (2010), the Polycentropodidae are divided into four diagnostic clusters, based primarily on wing venation and number of spurs on the legs: *Neureclipsis* cluster, *Polycentropus* cluster, *Cyrnus* cluster, and *Cyrnodes* cluster.

In contrast to the cosmopolitan extant Polycentropodidae, the findings of extinct polycentropodid species are confined to four amber deposits:

1. In Miocene Dominican amber, the family Polycentropodidae is represented by only two species of *Cernotina* (Wichard 2007; Wichard and Neumann 2021) which belong to the *Cyrnodes* cluster.
2. In Baltic Pleistocene amber (including findings in Saxonian Bitterfeld amber and Ukrainian Rovno amber), polycentropodids represent the dominant group of fossil caddisflies, accounting for well over 80% of all caddisfly specimens found. According to Ulmer (1912), these polycentropodids belong to 67 extinct species. More recently, several more species have been described from Pleistocene amber deposits (e.g., Mey 1986; Wichard et al. 2009; Ivanov and Melnitsky 2013; Wichard 2013; Melnitsky et al. 2021a, b), increasing the number of species to 118 (Morse 2022).

Most species belong to the genera *Holocentropus*, *Plectrocnemia*, and *Polycentropus* and are assigned to the *Polycentropus* cluster; other species belong to the genus *Nyctiophylax* of the *Cyrnus* cluster. The small *Neureclipsis* cluster includes only a few species, which are four *Neureclipsis* species and two *Archaeoneureclipsis* species which were transferred to the genus *Neureclispsis* by Oláh and Johanson (2010).

1. From Late Cretaceous Taymyr amber (Siberia, Russian Federation), six polycentropodid species have been described (Botosaneanu and Wichard 1983; Melnitsky and Ivanov 2022a, b). All belong to the extinct genus *Archaeopolycentra*, which cannot be assigned to any of the extant four diagnostic clusters, sensu Oláh and Johanson (2010).
2. In mid-Cretaceous Burmese amber, eight polycentropodid species have been found belonging to the *Neureclipsis* and *Polycentropus* clusters. Of these, five species are described in this paper.

## ﻿Materials and methods

The amber material was collected by local people in the Hukawng Valley of northern Myanmar, (Myitkyina District, Kachin State) and dates from the middle Cretaceous (Cenomanian) period about 98.8 ± 0.6 Ma ago (Shi et al. 2012), but the geological age of Burmese amber can be expected to be slightly older.

The Burmese amber with the embedded trichopteran inclusions was cut, face-grinded, and polished using a cutting machine and a polishing machine, a RotoPol-25 (Struers), with grinding paper for metallography: 800, 1200, 2500, and 4000 grit. Colour photographs were produced for the documentation of specimens. A Leica M420 macroscope with Apozoom 1:6 was used in combination with a Canon EOS 80D, EOS 3.0 utility software, and Zerene Stacker software. Measurements were made with a Leica SApo ocular micrometer.

Adult caddisflies embedded in amber are slightly flattened and visible in ventral and/or dorsal views. Very rarely forewings and hind wings are separately visible in amber inclusions. The wings are often saddle-roofed and cover the abdomen and genitalia in dorsal and lateral views. The genitalia are visible only in ventral or ventral-lateral views. Therefore, diagnoses and descriptions of male genitalia are usually limited to the ventrally located pair of inferior appendages alone.

Type-specimens in this study are deposited in the following repositories:

**NIGP**Nanjing Institute of Geology and Palaeontology, Nanjing, China

**ZFMK**Zoological Research Museum Alexander Koenig, Bonn, Germany

## ﻿Systematic palaeontology

### ﻿Order Trichoptera Kirby, 1813

**Suborder Annulipalpia Martynov, 1924**

**Superfamily Psychomyioidea Walker, 1852**

#### Family Polycentropodidae Ulmer, 1903

##### ﻿Genus Neureclipsis

Taxon classificationAnimaliaTrichopteraPolycentropodidae

McLachlan, 1864

4D8A809A-2083-5955-AED2-46A1A3F0ACA0

###### Type species.

*Phryganeabimaculata* Linnaeus, 1758.

###### Description and diagnosis.

Ocelli absent. Filiform antennae no longer than forewings. Maxillary palps each five-segmented with 1^st^ and 2^nd^ segments much shorter than 3^rd^ segment, terminal segment longest, annulated, and flexible. *Neureclipsis* species have complete wing venations with apical forks I, II, III, (IV), V on forewings and apical forks I, II, III, V on hind wings. In fore- and hind wings, fork I petiolate, fork II sessile, discoidal cell subtriangular, closed, crossvein *m* sloping. In forewings medial and thyridial cells usually present. Tibial spur formula 3/4/4.

*Neureclipsis* is distinguished from all other polycentropodid genera, except *Neucentropus*, by the presence of folk III in the hind wings. The following three new *Neureclipsis* species differ in their forewing lengths and in the number of their flagellomeres but are characterized especially by the male genitalia, clearly in the inferior appendages, which are one-segmented, long, and monolobed.

##### ﻿ Neureclipsis triangula sp. nov.

Taxon classificationAnimaliaTrichopteraPolycentropodidae

DB1BD3E5-0E26-58BF-8961-ADD0330BECE3

https://zoobank.org/8F917EDA-F2C2-4193-9AA2-C185281408EE

Fig. 1

###### Diagnosis.

The extinct species *Neureclipsistriangula* sp. nov. is characterized by a pair of slightly cupped inferior appendages running parallel in ventral view. Each appendage is tapered at the base, wider near the middle and apically forming a sub-triangular shape. Its sloping apical edge is clearly subapically toothed and highlighted in black.

**Figure 1.**
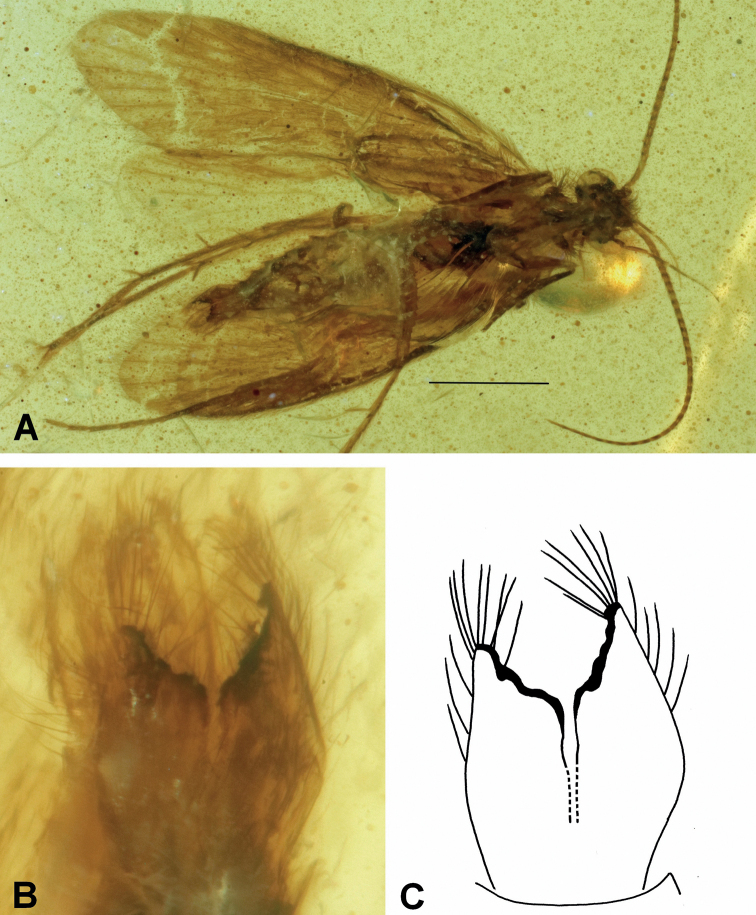
*Neureclipsistriangula* sp. nov. **A** male holotype (NIGP200021) habitus, ventral view **B** inferior appendages, ventral view **C** drawing of inferior appendages, ventral view. Scale bar: 1 mm.

###### Etymology.

Species named after the inferior appendages, subtriangular (Latin adjective = triangulus, -a, -um).

###### Holotype.

♂; Myanmar, Kachin State, Hukawng Valley; exact locality unknown; Mid-Cretaceous Burmese amber inclusion; deposited in the amber collection of the NIGP; NIGP200021.

###### Description.

Genus as described above. Body well preserved and visible in ventral and dorsal views. Forewing length about 4.2 mm, oblong, rounded, light brown. Antennae about two-thirds as long as forewings, with about 36 flagellomeres plus scapus and short pedicellus. Inferior appendages having subtriangular shape, with oblique, subapically toothed, and black terminal margin.

##### ﻿ Neureclipsis acuta sp. nov.

Taxon classificationAnimaliaTrichopteraPolycentropodidae

1A772395-7D78-5AC0-B312-939964E56C3F

https://zoobank.org/4C948C37-9772-4D7F-B9FB-15289400065F

Fig. 2

###### Diagnosis.

The extinct species *Neureclipsisacuta* sp. nov. is characterized by a pair of long inferior appendages tapering in the apical region and ending with a black, beak-shaped cap.

**Figure 2.**
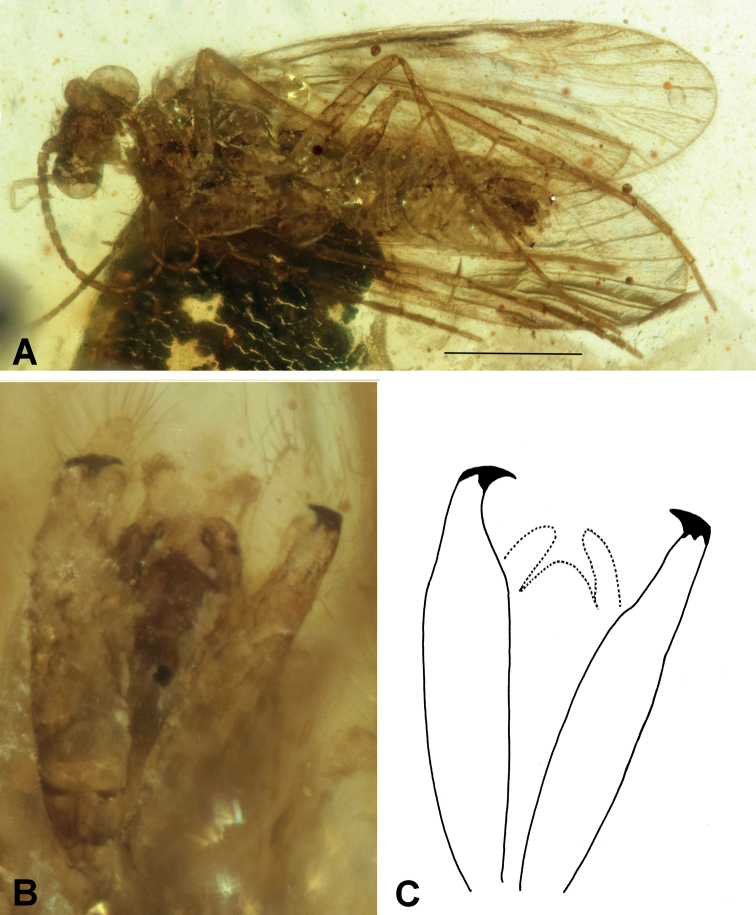
*Neureclipsisacuta* sp. nov. **A** male holotype (NIGP200022) habitus, ventral view **B** inferior appendages, ventral view **C** drawing of inferior appendages, ventral view. Scale bar: 1 mm.

###### Etymology.

Species named after the inferior appendages, apically sharpened (Latin adjective = acutus, -a, -um).

###### Holotype.

♂; Myanmar, Kachin State, Hukawng Valley; exact locality unknown; Mid-Cretaceous Burmese amber inclusion; deposited in the amber collection of the NIGP; NIGP200022.

###### Description.

Genus as described above. Body well preserved and visible in ventral and dorsal view, dorsum partially covered by dark artefacts. Forewing length about 3.0 mm, rounded, light brown. Antennae two-thirds as long as forewings with about 24 flagellomeres plus scapus and pedicellus. Inferior appendages long, bearing black, beak-shaped cap apically.

##### ﻿ Neureclipsis obtusa sp. nov.

Taxon classificationAnimaliaTrichopteraPolycentropodidae

7824CFF0-12E4-5902-8AF7-6495245590E6

https://zoobank.org/10D154EC-1DD9-40B0-A88E-F2285CE3FAA5

Fig. 3

###### Diagnosis.

The extinct species *Neureclipsisobtusa* sp. nov. has a distinctive pair of rod-shaped, long, inferior appendages. Apically, each appendage ends with an oblique oval surface, on which there a few, scattered stout bristles on the oval and a cluster of small setae on the edge of the oval.

**Figure 3.**
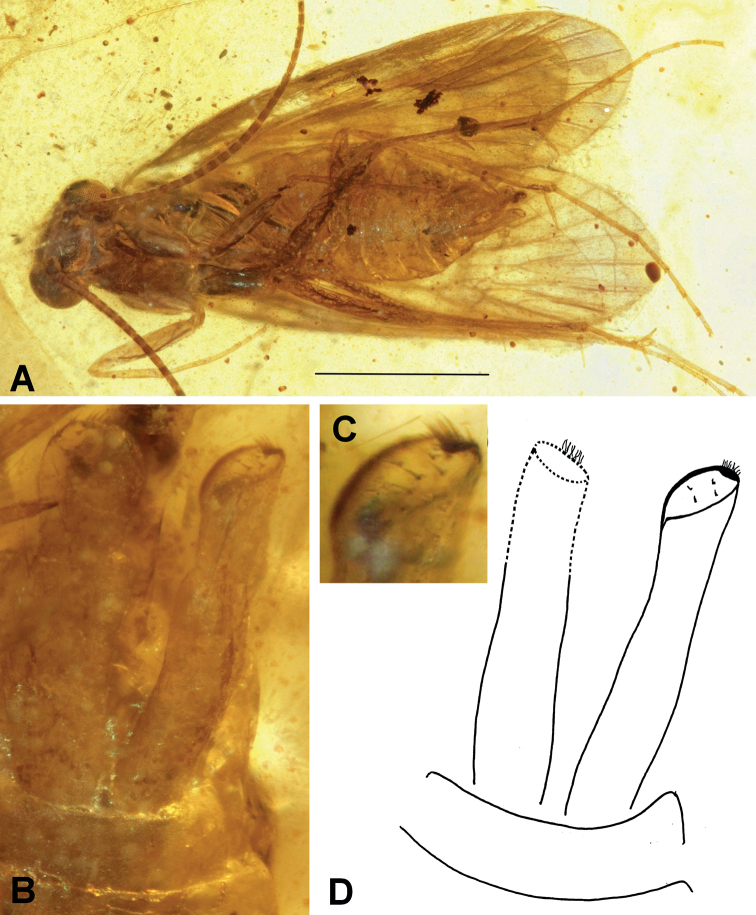
*Neureclipsisobtusa* sp. nov. **A** male holotype (NIGP200023) habitus, ventral view **B** inferior appendages, ventral view **C** apical oval surface of right inferior appendage, with small spines and cluster of setae, ventral view **D** drawing of inferior appendages, ventral view. Scale bar: 1 mm.

###### Etymology.

Species named after the inferior appendages, apically blunted (Latin adjective = obtusus, -a, -um).

###### Holotype.

♂; Myanmar, Kachin State, Hukawng Valley; exact locality unknown; Mid-Cretaceous Burmese amber inclusion; deposited in the amber collection of the NIGP; NIGP200023.

###### Description.

Genus as described above. Body well preserved and visible in ventral and dorsal views, dorsum slightly decomposed. Forewing length about 3.5 mm, broad and rounded, light brown. Antennae as long as forewings, with about 42 flagellomeres plus scapus and pedicellus. Inferior appendages long, parallel-sided, apically with oblique oval surface.

##### ﻿Genus Plectrocnemia

Taxon classificationAnimaliaTrichopteraPolycentropodidae

Stephens, 1836

AADF72F1-0B95-52EE-88C0-2A71A06458AB

###### Type species.

*Plectrocnemiasenex* Stephens, 1836.

###### Description and diagnosis.

Ocelli absent. Filiform antennae about as long as forewings or shorter. Maxillary palps each five-segmented with the 1^st^ and 2^nd^ segments much shorter than the 3^rd^ segment, terminal segment longest and annulated. *Plectrocnemia* adults with complete forewing venation, apical forks I, II, III, IV, V present; fork I petiolate and fork II sessile; discoidal and median cells closed; crossveins *r*, *s*, *r-m*, and *m* usually present. In hind wings apical forks I, II, V present, fork I petiolate and fork II sessile; discoidal cell closed. Tibial spur formula 3/4/4.

The two new *Plectrocnemia* species are very similar and differ clearly in the one-segmented inferior appendages which are robust and short or long.

##### ﻿ Plectrocnemia ohlhoffi sp. nov.

Taxon classificationAnimaliaTrichopteraPolycentropodidae

5DB42713-9C55-51AD-AFF0-5A05FAA3DD13

https://zoobank.org/6FE60CF6-492D-4E0D-A7E8-EB1535E863E6

Fig. 4

###### Diagnosis.

The extinct species *Plectrocnemiaohlhoffi* sp. nov. is characterized by a pair of elongate inferior appendages, slightly diverging distally and slightly curved apically toward each other. The appendages are weakly concave mesally along their length. In ventral view, each appendage is rounded at the apex and slightly concave in shape apicolaterally, each with a subapical tooth and a weakly projecting apicomesal corner.

**Figure 4.**
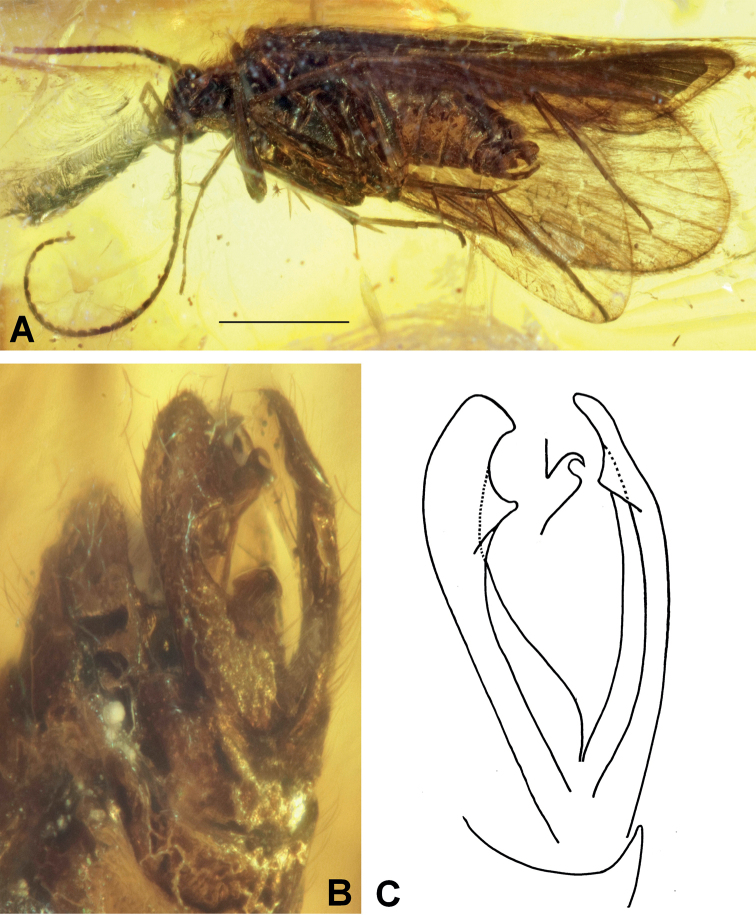
*Plectrocnemiaohlhoffi* sp. nov. **A** male holotype (ZFMK-TRI000834) habitus, ventral view **B** inferior appendages, left ventrolateral view **C** drawing of inferior appendages, left ventrolateral view. Scale bar: 1 mm.

###### Etymology.

The fossil species is dedicated to Rainer Ohlhoff, who donated the type specimen to the Zoological Research Museum Alexander Koenig, Bonn, Germany (ZFMK) for permanent preservation.

###### Holotype.

♂; Myanmar, Kachin State, Hukawng Valley; exact locality unknown; Mid-Cretaceous Burmese amber inclusion; deposited in the amber collection of the ZFMK; former Rainer Ohlhoff collection; ZFMK-TRI000834.

###### Description.

Genus as described above. Body well preserved, visible in left ventrolateral view. Forewing length about 4.2 mm. Forewings hyaline, light brown. Antennae about two-thirds as long as forewings with about 30 flagellomeres plus scapus and pedicellus; left antenna incomplete. Inferior appendages elongate, each forming an elongate shell and both inclining towards the genital midline.

##### ﻿ Plectrocnemia bowangi sp. nov.

Taxon classificationAnimaliaTrichopteraPolycentropodidae

4A764643-F9C2-53B4-894C-979FC5EE7573

https://zoobank.org/0A4CBE34-C7B9-425E-8C91-994D9B01AAB6

Fig. 5

###### Diagnosis.

The extinct species *Plectrocnemiabowangi* sp. nov. is characterized by a pair of spoon-like inferior appendages. On the inner side of each of the two spoon-shaped appendages there is a long needle, the tips of which touch each other in about the middle of the genital space.

**Figure 5.**
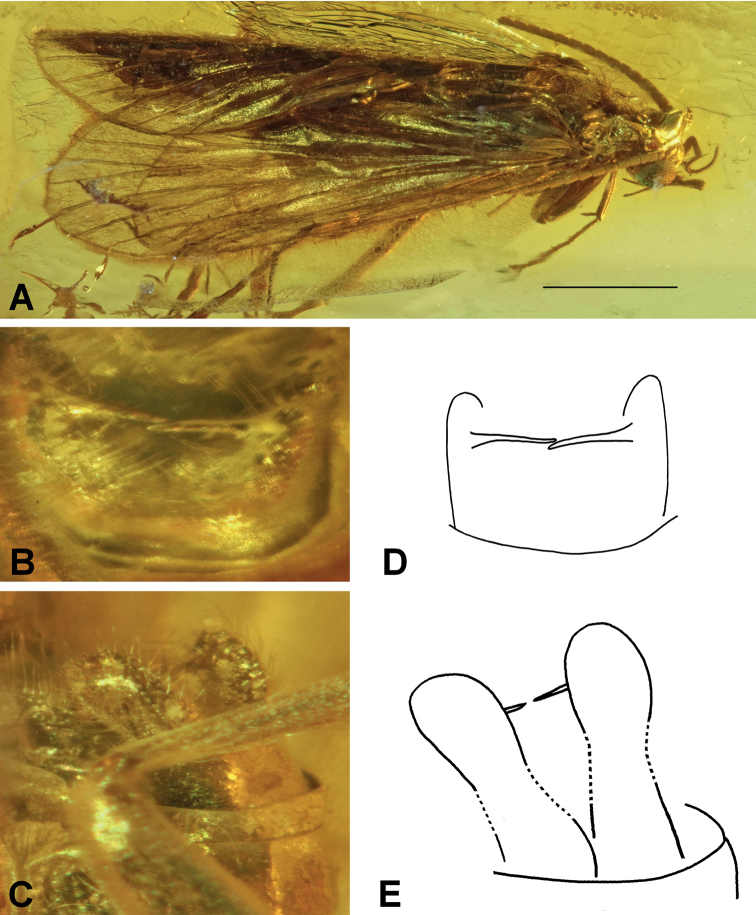
*Plectrocnemiabowangi* sp. nov. **A** male holotype (NIGP200024) habitus, dorsal view **B** inferior appendages, each with a long transverse needle on mesal surface, dorsal view **C** inferior appendages, in ventral view, covered by left hind leg **D** drawing of the long needles arising on the mesal surfaces of the inferior appendages, dorsal view **E** drawing of the pair of spoon-shaped inferior appendages, ventral view. Scale bar: 1 mm.

###### Etymology.

The fossil species is dedicated to Prof. Dr Bo Wang, Nanjing Institute of Geology and Palaeontology, China, who provided numerous Burmese ambers for taxonomic studies of embedded caddisflies.

###### Holotype.

♂; Myanmar, Kachin State, Hukawng Valley; exact locality unknown; Mid-Cretaceous Burmese amber inclusion; deposited in the amber collection of the NIGP; NIGP200024.

###### Description.

Genus as described above. Body well preserved, visible in ventrolateral view; right forewing visible in lateral view. Antennae incomplete, probably antennae about two-thirds as long as forewings. Forewing length about 4 mm. Forewings hyaline, light brown. Hind wing smaller than forewings, hyaline, light brown. Inferior appendages only partially visible in lateral view because covering by basal tarsus of left hind leg.

### ﻿Key of polycentropodid species in mid-Cretaceous Burmese amber

Family characters: ocelli absent. Five-segmented maxillary palps each with short 1^st^ and 2^nd^ segments and terminal segment longest and annulated. Forewing apical fork 1 petiolate; discoidal cell closed. Tibial spurs 3/4/4. Genital inferior appendages each one-segmented.

**Table:** 

1	Forewing forks I, II, III, (IV), V, hindwings with fork III (*Neureclipsis* cluster)	**2**
–	Forewing forks I, II, III, IV, V, hindwings without fork III (*Polycentropus* cluster)	**7**
2	Forewing apical forks I, II, III, V, but fork IV absent	***Electrocentropusdilucidus***
–	Forewing apical forks I, II, III, IV, V	**3**
3	hind wings apical forks II, III, V	***Neucentropusmacularis***
–	hind wings apical forks I, II, III, V	**4**
4	Inferior appendages long and slim	**5**
–	Inferior appendages subtriangular	***Neureclipsistriangula***
5	Apices of inferior appendages subapically ampullate	***Neurecipsisburmanica***
–	Apices of inferior appendages straight	**6**
6	Apices of inferior appendages beak-shaped, black	***Neureclipsisacuta***
–	Apices of inferior appendages forming oval plate	***Neureclipsisobtusa***
7	Inferior appendages forming long, narrow bowl	***Plectrocnemiaohlhoffi***
–	Inferior appendages spoon-shaped, each with thin long transverse needle arising on mesal surface	***Plectrocnemiabowangi***

## ﻿Conclusions

Polycentropodids are found only sporadically in Burmese amber. This fact is especially true in comparison to the numerous polycentropodid specimens in Eocene Baltic amber, which belong to the *Polycentropus* cluster and its genera *Plectrocnemia*, *Polycentropus*, and *Holocentropus*, and also to the genus *Nyctiophylax* in the *Cyrnus* cluster (Ulmer 1912; Wichard et al. 2009).

Species of the *Neureclipsis* cluster predominate in the Burmese Amber. The cluster includes the genus *Neucentropus* with an extinct amber species (*N.macularis*) and an extant species that now occurs in southern Russian Far East, Mongolia, China, Vietnam, and Japan (Li et al. 1998; Morse et al. 2019), and also an extinct amber genus *Electrocentropus* (*E.dilucidus*) with the characteristically absent fork IV of the forewings (Wichard 2021), as well as the genus *Neureclipsis* of which four fossil species currently are known to occur in Burmese amber.

Additional *Neureclipsis* species are expected in the near future, as some amber inclusions indicate the *Neureclipsis* cluster, but the limited state of preservation of amber does not always allow for careful taxonomic analysis and description.

## Supplementary Material

XML Treatment for Neureclipsis

XML Treatment for Neureclipsis triangula

XML Treatment for Neureclipsis acuta

XML Treatment for Neureclipsis obtusa

XML Treatment for Plectrocnemia

XML Treatment for Plectrocnemia ohlhoffi

XML Treatment for Plectrocnemia bowangi
